# Effect of Nickel as Stress Factor on Phenol Biodegradation by *Stenotrophomonas maltophilia* KB2

**DOI:** 10.3390/ma14206058

**Published:** 2021-10-14

**Authors:** Agnieszka Gąszczak, Elżbieta Szczyrba, Anna Szczotka, Izabela Greń

**Affiliations:** 1 Institute of Chemical Engineering, Polish Academy of Sciences, ul. Bałtycka 5, 44-100 Gliwice, Poland; eszczyrba@iich.gliwice.pl (E.S.); szlemp@iich.gliwice.pl (A.S.); 2 Faculty of Natural Sciences, Institute of Biology, Biotechnology and Environmental Protection, University of Silesia in Katowice, ul. Jagiellońska 28, 40-032 Katowice, Poland; Izabela.Gren@us.edu.pl

**Keywords:** heavy metal inhibition, kinetic equations, nickel, phenol biodegradation

## Abstract

This study focuses on the phenol biodegradation kinetics by *Stenotrophomonas maltophilia* KB2 in a nickel-contaminated medium. Initial tests proved that a nickel concentration of 33.3 mg·L^−1^ caused a cessation of bacterial growth. The experiments were conducted in a batch bioreactor in several series: without nickel, at constant nickel concentration and at varying metal concentrations (1.67–13.33 g·m^−3^). For a constant Ni^2+^ concentration (1.67 or 3.33 g·m^−3^), a comparable bacterial growth rate was obtained regardless of the initial phenol concentration (50–300 g·m^−3^). The dependence *µ* = f (*S*_0_) at constant Ni^2+^ concentration was very well described by the Monod equations. The created varying nickel concentrations experimental database was used to estimate the parameters of selected mathematical models, and the analysis included different methods of determining metal inhibition constant *K_IM_*. Each model showed a very good fit with the experimental data (R^2^ values were higher than 0.9). The best agreement (R^2^ = 0.995) was achieved using a modified Andrews equation, which considers the metal influence and substrate inhibition. Therefore, kinetic equation parameters were estimated: *µ_max_* = 1.584 h^−1^, *K_S_* = 185.367 g·m^−3^, *K_IS_* = 106.137 g·m^−3^, *K_IM_* = 1.249 g·m^−3^ and *n* = 1.0706.

## 1. Introduction

Research conducted in recent years has confirmed that the environment is polluted with organic compounds and heavy metals. The aliphatic hydrocarbons, mono and polycyclic aromatic hydrocarbons together with nickel, zinc, cadmium or mercury are present in surface waters and soil. These pollutants usually have dissimilar physicochemical properties, making their removal a very tedious process [1,2,3,4,5]. Among soil and wastewater pollutants, phenol and nickel are one of the most common. Phenol is an aromatic hydrocarbon, one of 126 chemical contaminants registered by the United States Environmental Protection Agency [6]. Due to the enormous scale of this basic organic raw material use, phenol occurs in the air, water, soil and bottom sediments. Together with its derivative compounds, it can be found in many sorts of different industries such as petrochemical, pharmaceutical, textile and plastic industries, paint manufacturing, paper and pulp factories, etc. [1]. The production facilities in these industries very often have to deal with the presence of phenol in their wastewaters, at concentrations ranging from 1 to 7000 mg·L^−1^ [7]. Among the various types of effective phenol removal technologies, biological decomposition of hazardous substances attract attention with their effectiveness and low economic costs [8,9,10,11,12,13]. Despite being toxic, phenol can be utilized by microbes as carbon and energy sources [8,14,15,16,17]. Undoubtedly, the efficiency of its removal will be significantly influenced by the amount of phenol, but also the environmental conditions and the presence of other organic or inorganic compounds.

Heavy metals in multiple industrial, agricultural, medical and technological applications have led to their wide distribution in the environment. Nickel is one of the elements necessary for the growth of microorganisms and plays essential roles in various microbial cellular processes when incorporated into nickel-dependent enzymes (acetyl CoA decarbonylase/synthase, urease, methylenediurease, Ni-Fe hydrogenase, carbon monoxide dehydrogenase, methyl coenzyme reductase) [18]. In excessive amounts, it becomes toxic to microorganisms, which in turn leads to inhibition of the bacteria growth and decomposition of organic compounds [19,20]. Four mechanisms of nickel toxicity are distinguished: (a) Ni replaces the essential metal of metalloproteins, (b) Ni binds to catalytic residues of non-metalloenzymes, (c) Ni binds outside the catalytic site of an enzyme to inhibit allosterically, (d) Ni indirectly causes oxidative stress [18,21,22]. However, bacteria have developed different metal resistance strategies: modifications of the cell sheaths to prevent the penetration of metal ions into the cytoplasm (modification of the permeability of the cell sheaths), removal of toxic metal ions from the cytoplasm outside the cell (extracellular or non-cellular ion binding by bacterial metabolites), enzymatic detoxification of metal ions, intracellular ion binding [23,24].

Phenol and nickel are often found together in industrial spots and wastewaters caused by anthropogenic activities such as metal plating or dye and painting industries [25]. Furthermore, production waste of chemical plants in the form of phenolized sludge, tar waste and other hazardous by-products create huge heaps that contaminate the surrounding environment. Such a situation occurred in Poland in the Śląskie Voivodeship. Water tests of the Kalina pond showed that, among others, acceptable standards of pH, arsenic, chromium, zinc, nickel, phenols and aromatic hydrocarbons were exceeded [26].

The influence of nickel presence on biodegradation process was not extensively studied and clearly defined. It was the subject of a few studies only, and their authors inform about the various effects of the co-exposure of hydrocarbons and this metal. Asitok et al. [19] found that nickel concentration of 1.17 mM significantly reduced the specific growth rate of *Pseudomonas fluorescens* (from 0.447 h^−1^ to 0.186 h^−1^) and *Vibrio fluvialis* (from 0.443 h^−1^ to 0.185 h^−1^). Doubling the metal concentration completely inhibited the growth of both strains. Some strains, e.g., *Arthrobacter bambusae* AQ5-003 or *Glutamicibacter,* are reported to have the capability of utilizing phenol in 100% although the addition of nickel salt into the phenol media [27,28]. *Acinetobacter* sp. also showed the ability to utilize phenol in the presence of nickel; furthermore, the biodegradation time was significantly shortened (from 48 to 18 h) after the cell immobilization in gellan gum beads [29]. The effect of heavy metals on *Cobetia* sp. SASS1 strain cells growth and phenol degradation were investigated at the optimal pH and salinity. Comparing with the control group, the OD600 value decreased remarkably but phenol degradation efficiencies increased from 4.7 to 10.2% while biodegradation time was lengthened from 10 to 40 h [30]. Other researchers revealed a strong inhibition effect of nickel during phenol biodegradation by *Pseudomonas* sp. AQ5-04 or fungal strain *Debaryomyces* JS4 [31,32].

In the case of other hydrocarbons, the presence of Ni^2+^ ions caused: acceleration of the toluene degradation by P1 and P19 strains [33] and inhibition of the crude oil or phenanthrene biodegradation [20,34,35,36,37]. The bacteria: *Pseudomonas aeruginosa* CA207Ni, *Burkholderia cepacia* AL96Co and *Corynebacterium kutscheri* FL108Hg grew in hydrocarbon media amended with nickel and cobalt (at 5.0 mM) without any changes due to metal toxicity. It is noteworthy that Ni concentration appreciably reduced from 5.0 to 0.12–0.13 mM during described petroleum hydrocarbons biodegradation [38]. In another environment, the contamination with crude oil and nickel reduced the share dominant Proteobacteria and increased the abundance of Actinobacteria. The shift between Proteobacteria and Actinobacteria was beneficial to the removal of the organic pollutant [39]. Significant factors determining the course of microbial transformation are metal concentration and molecular weight of the compound, which could be due to their physical and chemical properties, which are a function of the number of rings in their structure [20,40].

To obtain the maximum efficiency of microbial revitalization of industrial areas or wastewater treatment, it is necessary to select microorganisms that can retain high activity in this peculiar environment. The *Stenotrophomonas maltophilia* KB2 is a strain with great biodegradation potential; however, the presence of nickel salts in industrial wastewaters containing phenol can have a significant impact on the rate and efficiency of biodegradation process. Co-contamination by phenol and nickel is a complex problem because the joint occurrence of these harmful pollutants results in an appearance of two types of inhibition: substrate inhibition and metal inhibition.

The aim of this work was to evaluate efficiency of phenol degradation under various nickel ion concentrations and determine the lowest concentration of nickel that will inhibit the visible growth of a microorganism. The overriding goal was to develop a kinetic equation taking into account the influence of the introduced metal on specific growth rate and develop methodology for determining the parameters of this equation.

## 2. Materials and Methods

### 2.1. Chemicals and Mediums

The composition of mineral salts medium (MSM) for bacteria culturing was: Na_2_HPO_4_·12H_2_O 3.78 g; KH_2_PO_4_ 0.5 g; NH_4_Cl 5 g; MgSO_4_·7H_2_O 0.2 g; yeast extract 0.01 g; deionized water 1000 mL; enriched with TMS (Trace Mineral Solution), in the amount of 1 mL per 1000 mL of MSM. TMS contained: FeSO_4_·7H_2_O 3.82 g; CoSO_4_·7H_2_O 0.3 g; MnSO_4_·H_2_O 0.08 g; ZnSO_4_·7H_2_O 0.14 g; H_3_BO_3_ 0.006 g; Na_2_MoO_4_·2H_2_O 0.04 g; NiSO_4_·7H_2_O 0.08 g; CuSO_4_·5H_2_O 0.003 g; Na_2_WO_4_·2H_2_O 0.006 g dissolved in 10 mL of 32% HCl, Al_2_(SO_4_)_3_·18H_2_O 0.15 g and 1000 mL deionized water. The ability of the KB2 strain to utilize phenol in the presence of Ni^2+^ ions was tested by the inoculation of the strain in a modified MSM; Na_2_HPO_4_·12H_2_O and KH_2_PO_4_ content was reduced to one-third. The metal solution was prepared by soluble 23.9 g of NiSO_4_·7H_2_O in 50 mL of the deionized water.

### 2.2. Chemical Analysis

The changes of substrate concentration in the liquid culture were determined by a Waters HPLC equipped with Waters 1525 gradient pump and two-wave detector UV-VIS Waters M2487 (Milford, CT, USA). The separation was carried out on reverse phase column (Spherisorb ODS 2.5 μm, 150 mm × 4.6 mm). The mobile phase was the system methanol and 1% acetic acid (40:60 *v/v*) with the flow rate of 1 mL·min^−1^; detection was carried out at wave length 272 nm. Samples were taken from the bioreactor and subjected to centrifugation (12,000 rpm, 15 min.), next filtration (0.22 μm pore diameter syringe filters) and diluted with deionized water before chromatographic analyses. Bacterial cells density was determined spectrophotometrically (spectrophotometer HACH DR3900, Dusseldorf, Germany) by measuring the absorbance at a wavelength *λ* = 550 nm. Before the kinetics studies, the calibration curve was prepared which made it possible to convert optical density into grams of dry weight mass.

### 2.3. Microorganisms

The *Stenotrophomonas maltophilia* KB2 strain was obtained from the collection of Institute of Biology, Biotechnology and Environmental Protection, Faculty of Natural Science of the University of Silesia in Katowice, Poland. It was isolated from activated sludge of the sewage treatment plant in Bytom—Miechowice in Poland what was described previously [41]. Now, it is also stored under number E-113197 in VTT Collection in Finland. These Gram-negative bacteria was found to utilize different aromatic substrates as sole carbon and energy source, e.g., phenol, catechol, benzoic acid, protocatechuic acid, 4-hydroxybenzoic acid and vanillic acid. Depending on the inductor structure three various dioxygenases could be induced in these strain cells [41].

It is known from previous studies that the KB2 strain has the ability to degrade phenol at a concentration of at least 940 g·m^−3^. During aerobic phenol biodegradation in the first stage monooxygenase [EC 1.14.13.7] catalyses the attachment of a hydroxyl group to an aromatic ring to form catechol [42]. Subsequently, degradation takes place via the meta route, with the end product being acetaldehyde and pyruvic acid. This pathway was confirmed by the detection of catechol 2,3-dioxygenase activity (4.92 ± 0.34 µmol·min^−1^·mg of protein^−1^) after phenol cell induction.

Phenolic monooxygenase, isolated from *S. maltophilia* KB2 cells, lost its activity after adding copper sulphate or iron chloride. Tests on the sensitivity of catechol 2, 3-dioxygenase demonstrated that activity of this enzyme slightly decreased in the presence of Ni2+ ions [43]. The comparison of the available data on phenol-degrading strains with the results of our experiments carried out earlier showed that the *S. maltophilia* KB2 strain is characterized by a short stagnation phase, high biodegradation activity (fast phenol utilization) and optimal growth rate achieved at relatively high phenol concentrations.

In order to prepare microorganisms for the kinetics studies, *S. maltophilia* KB2 cells were transferred from the agar slopes to the tubes containing 20 mL of sterile mineral salts medium supplemented with 5.65 mg of phenol. After a 24 h incubation (130 rpm, 30 °C) the cell suspension was placed in the sterile Erlenmeyer flask, filled up to a volume of 100 mL with mineral medium and 28.2 mg of phenol. In the next day the freshly proliferated cells were transferred to 500 mL sterile Erlenmeyer flasks, mineral salts medium was added to obtain the culture volume of 300 mL and 84.7 mg of phenol was introduced. Culture flasks were kept on an orbital shaker at 130 rpm at 30 °C for 72 h, and every 24 h a new dose of phenol was applied. After that, the cells were harvested by centrifugation (4500× *g*, 15 min, 4 °C), washed with sterile mineral medium and after being suspended in 20 mL mineral medium were kept at 4 °C up to 14 days.

### 2.4. Biodegradation Experiments

Tests were carried out in the 500 mL Erlenmeyer flasks and bioreactor with the working volume of 1.5 dm^3^ (Biostat B, Sartorius, Melsungen, Germany). The flask cultures were shaken on an orbital shaker at 130 rpm at 30 °C. The equipment of the bioreactor (temperature, pH and O_2_ sensors) made it possible to maintain optimal environmental conditions—30 °C, pH = 7 and stirrer rotations 300 rpm. The concentration of dissolved oxygen (DO) in the suspension was kept about 5 mg·dm^−3^ through aeration by external compressor. Each cultivation (both in the flasks and in the bioreactor) was started with the similar cell concentration in the solution, equaling 61.3 g_dcw_·m^−3^ (the initial OD = 0.2). Phenol and nickel salt were added as a solution (37.6 g·dm^−3^ and 478 g·dm^−3^, respectively). The cultures were sampled at regular time intervals and both absorbance and phenol concentration were determined.

### 2.5. Modelling Kinetics of Cell Growth on Phenol

The process of organic compound utilization by bacteria is closely related to the growth of microorganisms that include this compound in their metabolic pathway. Cases of utilization of an organic compound without biomass growth are very rare, hence mathematical models of the kinetics of organic compounds biodegradation are based on the specific growth rate.

Phenol is a compound that inhibits the growth of the bacterial population. Due to mathematical simplicity Andrews model is extensive use for describing the growth on inhibitory substrates such as phenol [16,27,30]. The inhibitory growth kinetic equation is as follows:

(1) $$\mu =\frac{\mu_{max}\times S}{K_{s}+S+\frac{S^{2}}{K_{Is}}}$$

where *μ* is the specific growth rate, *S* is the substrate concentration, *K_s_* is the half saturation constant and *Ki* is the substrate inhibition constant. The *μ_max_* value in Equation (1) called the maximum specific growth rate is a parameter of this equation. The true maximum specific growth rate can be determined from Equation (2) [44,45]:

(2) $$\mu_{max}{}^{*}=\frac{\mu_{max}}{1+2\sqrt{\frac{K_{s}}{K_{Is}}}}$$

### 2.6. Modelling Kinetics of Cell Growth on Phenol in the Presence of Metal

The use of metal inhibition models to evaluate the effect of heavy metal ions presence to the bacterial growth rate is poorly represented in the literature despite the importance of such study. Several mathematical models taking into account the influence of heavy metals on the biodegradation of toxic substances were found in the available publications. They are mostly extensions of the Monod or Andrews equations [46,47,48].

The Han and Levenspiel model (Equation (1)) was introduced as an extension of the Monod equation and it includes inhibition effects that are present due to high concentration of substrate, cells or other products [49]. H-L model assumes that there is a critical inhibitor concentration where the growth rate slows down or the reaction ceases if that point is reached. This model is capable of explaining the types of inhibition as either competitive or non-competitive:

(3) $$\mu =\frac{\mu_{max}\times S}{K_{s}{\left(1-\frac{I}{I^{*}}\right)}^{m}+S}{\left(1-\frac{I}{I^{*}}\right)}^{n}$$

where I* is the critical inhibitor concentration, and *n* and *m* are constants. The inclusion of the parameter I* increased the model’s ability to predict cell growth in both toxic and non-toxic concentration.

The modified version of this model was used in the publications on the biodegradation of organic compounds in an environment containing heavy metals:

(4) $$\mu =\mu_{max}{\left(1-\frac{M}{M_{crit}}\right)}^{n}$$

where, *M* is the metal concentration, *M_crit_* is the critical metal concentration [50,51,52]. Kai et al. [53] used the Equation (5) to describe the growth of *Rhodococcus* sp. on diesel fuel in the presence of different concentrations of zinc:

(5) $$\mu =\mu_{max}e^{-kM}$$

The specific growth rate expressed with considering the substrate inhibition and heavy metal ion inhibition can be written as:

(6) $$\mu =\frac{\mu_{max}\times S}{K_{s}+S+\frac{S^{2}}{K_{IS}}}\times \frac{K_{IM}}{K_{IM}+M^{n}}$$

where *K_IS_* represents substrate inhibition constant, *K_IM_* represents heavy metal inhibition constant, *n* is an equation parameter.

The first factory of this equation accounts for substrate inhibupition. The constants $\mu_{max},$ $K_{s}$ and $K_{IS}$ can be determined after carrying out a series of measurements for various concentrations of the substrate. The second term takes into account metal inhibition. Constants $K_{IM}$ and *n* can be read from the graph $\log\left[\frac{\mu_{max}\times S}{\mu \left(K_{s}+S+\frac{S^{2}}{K_{IS}}\right)}-1\right]=f\left(\log M\right)$ on which the results of experiments conducted for constant substrate concentration and various metal concentrations are plotted. The experimental data points should follow the straight line, the slope of which is equal to “*n*” and the intercept is equal to log *K_IM_* [46].

Amor et al. used a form of the Andrews equation (originally used to describe substrate inhibition of microbial growth) to model the effect of cadmium, zinc and nickel on rates of alkyl benzene biodegradation [47]. If we assume the value *n* = 1 then, in accordance with the suggestion given by Amor et al., the heavy metals inhibition constant (*K_IM_*) could be calculated according to the equation:

(7) $$\mu =\frac{\mu_{max}\times M}{K_{s}+M+\frac{M^{2}}{K_{I}}}$$

Since only inhibitory effects were observed in the heavy metal concentration range tested, the equation could be simplified by considering that *K_s_* was negligible:

(8) $$\frac{1}{\mu }=\frac{1}{\mu_{max}}+\frac{M}{K_{IM\;}\times \mu_{max}}$$

In such cases, a plot of (specific growth rate)^−1^ versus heavy metal concentrations should yield a straight line.

## 3. Results and Discussion

### 3.1. Phenol Degradation by Stenotrophomonas Maltophilia KB2

Before starting the studies related to the kinetics of the microbial degradation of phenol, some preliminary tests were performed. It was found that the introduction of nickel ions into the standard KB2 culture causes the precipitation of phosphate salts. The resulting sediment disturbed the absorbance measurement and moreover, by binding some of the nickel ions, it reduced their concentration in the medium. There is no uniform approach to this issue in the publications devoted to the research on the heavy metals influence on the hydrocarbon’s biodegradation. Media containing the minimal concentration of PO_4_^3−^ ions [46,54] as well as media with a much higher phosphates concentration than in our MSM [9,10,29] were used. Due to the characteristics of the strain KB2 used, the pH could not be lowered too much. Therefore, the content of Na_2_HPO_4_·12H_2_O was reduced to 1.26 g·L^−1^ and KH_2_PO_4_ to 0.167 g·L^−1^. In such a modified MSM medium, no precipitation was observed, and the concentration of nickel ions remained constant.

**Figure 1:**
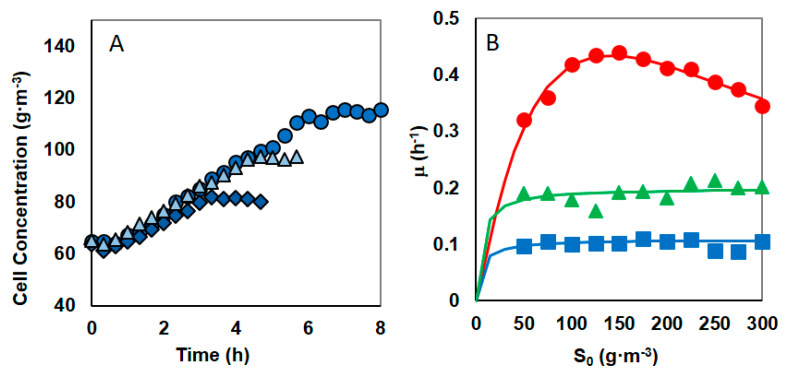
(**A**) The effect of the nickel presence (3.33 g·m^−^^3^) on the biomass growth for different initial phenol concentrations of 100 g·m^−3^ (diamonds), 200 g·m^−3^ (triangles) and 300 g·m^−3^ (circles).(**B**) Dependence of the growth rate on the initial phenol concentration in cultures without of nickel (red circles) and in cultures containing nickel at concentration 1.67 g·m^−^^3^ (green triangles) or 3.33 g·m^−^^3^ (blue squares).

The kinetics of the phenol biodegradation by *S.maltophilia* KB2 had been already known, but due to the change of mineral salt solution composition, the tests had to be repeated. The new studies on the phenol biodegradation kinetics by KB2 cells were conducted for initial phenol concentration changed within the range of 50–300 g·m^−3^. During experiments, in particular time intervals, the changes in concentration of biomass and growth substrate were detected, and next the *lnX =* f(*t*) graph was prepared. In the phase of exponential growth, the graph of this function is a straight line whose slope determines the value of the specific growth rate *µ.* The obtained experimental database *µ =* f(*S*_0_) (Figure 1, red circles)*,* made the selection of the kinetic model and the estimation of its parameters possible. For the substrate concentrations exceeding 175 g·m^−3^ the negative effect of phenol on bacterial growth was observed, so for the description of the process kinetics the Andrews model was applied.

Time course assay of *S. maltophilia* KB2 revealed that degradation of phenol at concentration range of 50 to 300 g·m^−3^ was reached within 1.5 and 5.5 h, respectively. Andrews model is well fitted with experimental data. The values of the kinetic equation parameters were estimated to give: *µ_m_* = 1.584 h^−1^, *K_s_* = 185.4 g·m^−3^, *K_IS_* = 106.1 g·m^−3^ (Nonlinear Regression Analysis program—NLREG (Sherrod 2010)). Here, *µ_m_* is one of the fitting parameters of the Andrews model, the true maximum growth rate (μ*_max_) was calculated from Equation (2) and it is equal 0.43 h^−1^.

Presented in recent publications parameters of the Andrews equation estimated for activated sludge and some phenol-degrading strains were listed in the Table 1. Among the compared strains, *S. maltophilia* KB2 strain is distinguished by the lowest substrate inhibition constant (*K_IS_* = 106.1) and the highest value of the true maximum growth rate (µ_max_* = 0.43).

### 3.2. Different Phenol Concentrations and Constant Nickel Concentration

#### 3.2.1. Heavy Metal Tolerance Determination

The tested strain showed a high sensitivity to the presence of nickel, even at its low concentration. Due to the toxicity of Ni^2+^, KB2 could not grow with a nickel concentration higher than 33.3 g·m^−3^ (0.567 mM). Bacterial growth was negligible relative to inoculum size and could not be detected as a significant increase. Interestingly, the chromatographic analysis samples taken after 24 h, showed complete degradation of the phenol. This result was in harmony with those mentioned by El-Deeb who found the MIC of nickel for *Pseudomonas putida* strains in the presence of phenol equal 0.6 mM [58]. Other researchers reported values from 0.5 mM to 6 mM [59,60,61,62].

#### 3.2.2. Constant Nickel Concentration

The experiments for initial phenol concentration changed within the range of 50–300 g·m^−3^ were repeated in two series in the presence of nickel at concentration 1.67 or 3.33 g·m^−3^. When phenol and nickel were present in the system, then at a constant nickel concentration, the increase in the phenol concentrations has a negligible impact on the specific growth rate. This is shown in Figure 1, which presents the course of 3 cultures at the same nickel concentration but differing in the amount of introduced phenol (Figure 1A) and specific growth rate for the initial phenol concentration in the range 50–300 g·m^−^^3^ (Figure 1B green triangles or blue squares). The substrate inhibition is imperceptible, so the Monod equation is appropriate for describing the specific growth rate as a function of initial phenol concentration. The values of the Monod equations parameters for the cultures with the same nickel content, were estimated using the least—square error method with the help of Nonlinear Regression Analysis program—NLREG (Sherrod 2010)), and they are presented in the Table 2. Some specific growth rate decrease might have been expected based on the results of earlier studies on the KB2 strain enzymes’ sensitivity to nickel. The presence of Ni^2+^ ions caused a decrease in the activity of 2.3 catechol dioxygenase by 86% [43].

### 3.3. Different Nickel Concentrations and Constant Phenol Concentration

**Figure 2:**
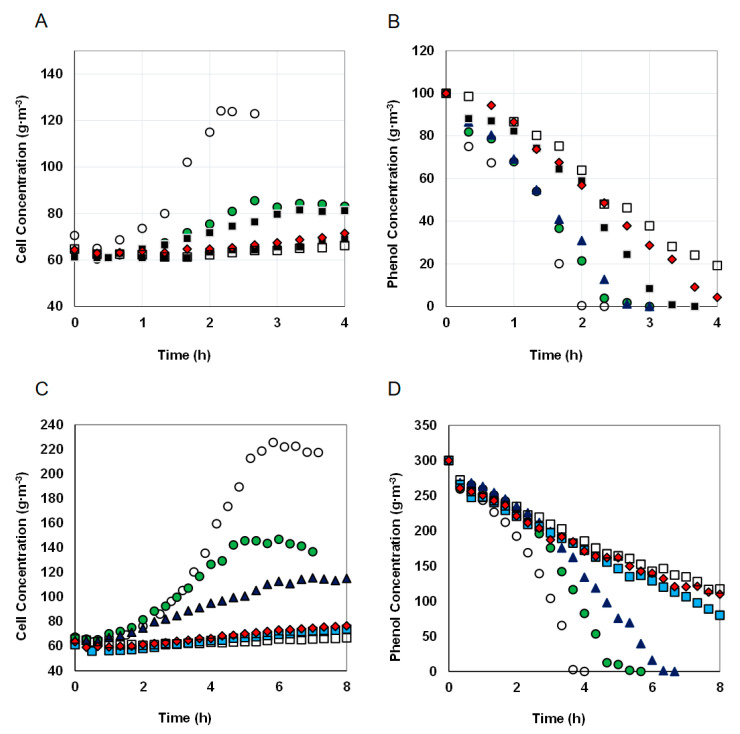
The effect of the nickel concentrations on the biomass growth and phenol utilization in two series of cultures for different initial phenol concentrations of 100 (**A**,**B**) and 300 g·m^−3^ (**C**,**D**). Nickel concentrations, g·m^−3^: 0 (○), 1.67 (●), 3.33 (▲), 6.67 (■), 10 (♦), 13.33 (□).

As presented in Figure 2, addition of nickel to MSM solution containing phenol noticeably changed the run of the process in a batch reactor. Regardless of the phenol concentration, the biomass growth rates and the biomass concentrations achieved at the end of batch cultures decreases with the increase in nickel concentration. The phenol decomposition process slowed down too. Substrate consumption took 1.7 h at the concentration of 100 g·m^−3^. The presence of nickel extended time needed to biodegradation of the same phenol dose to 3 h for Ni^2+^ concentration of 1.67 and to 5.33 h for Ni^2+^ concentration of 13.33 g·m^−3^.

Three series of tests were carried out for the nickel concentration range from 1.67 to 13.33 g·m^−3^ at three different initial phenol concentrations—100, 150 or 300 g·m^−3^. The phenol alone biodegradation tests showed that at *S*_0_ = 300 g·m^−3^ the substrate inhibition effect is observable, while 100 g·m^−3^ is the phenol concentration at which the specific growth rate is close to the maximum value.

**Figure 3:**
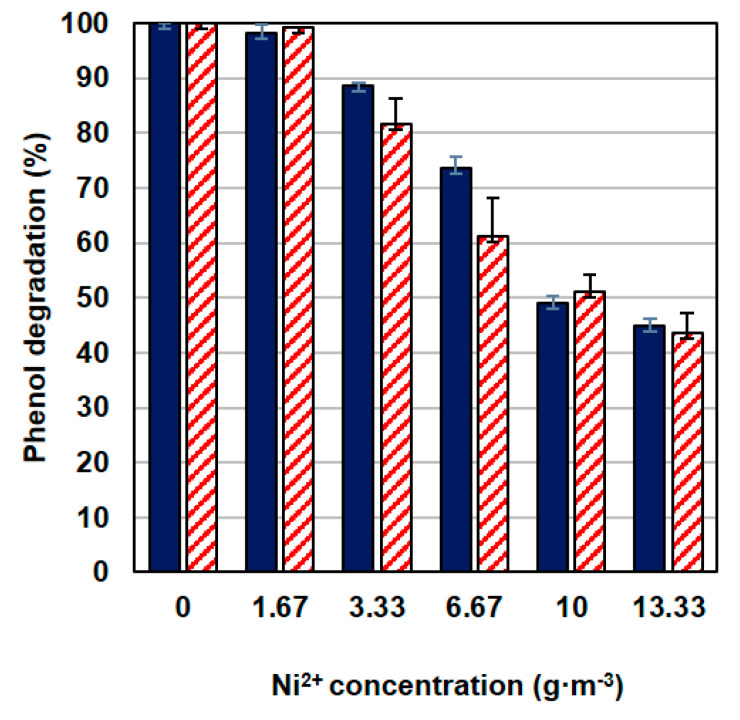
The effect of nickel concentration on phenol degradation at 100 g·m^−3^ (dark blue bars) and 300 g·m^−3^ (red pattern bars).

As the nickel concentration increased, the efficiency of phenol degradation decreased (Figure 3), a similar relationship applied to the increase in biomass (Figure 2A,C). The effectiveness of this process was not influenced by the phenol concentration, the differences in the systems containing different doses of phenol were small. One hundred g·m^−3^ of phenol was utilized in 100% by the KB2 strain within 140 min. At the same time period, the presence of nickel caused a decrease in the percentage of degraded phenol to 45% for metal concentration *M* = 13.33 g·m^−3^. Similarly, when the initial phenol concentration was 300 g·m^−3^ phenol was degraded in 100% within 300 min but in the presence of nickel at a concentration of 13.33 g·m^−3^ 43% of the substrate was decomposed at the same time.

### 3.4. Modelling the Specific Growth Rate as a Function of Different Nickel Concentrations

**Figure 4:**
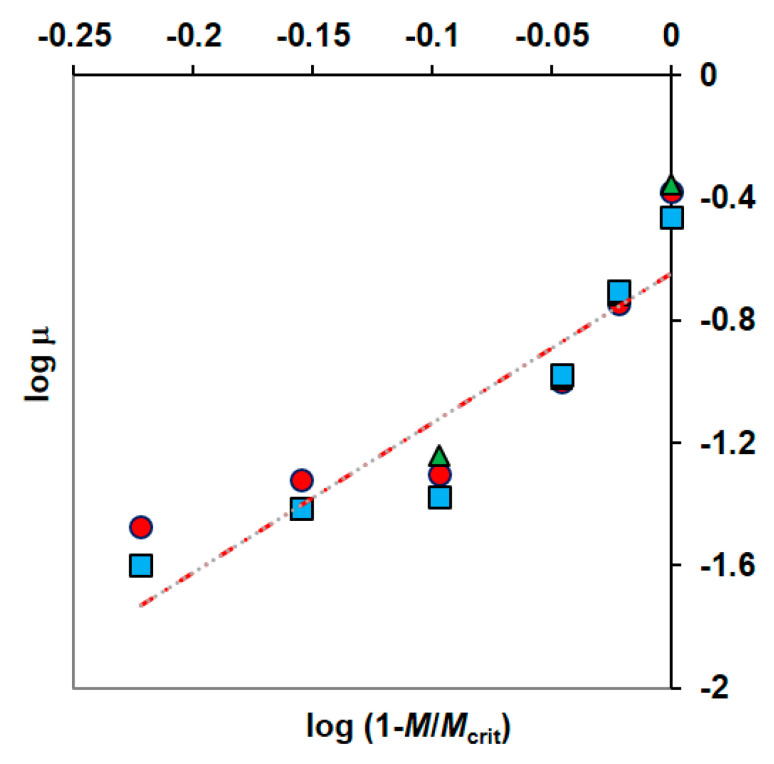
The method of determining the parameters of the Han-Levenspiel model (phenol concentrations: 100 g·m^−3^ (●), 150 g·m^−3^ (▲), 300 g·m^−3^ (■)).

**Figure 5:**
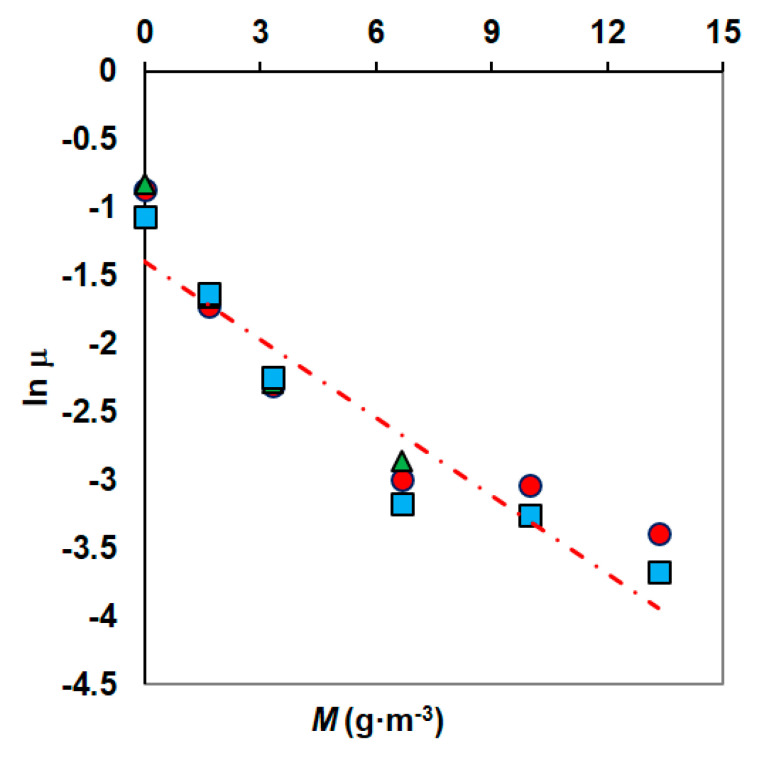
The method of determining the parameters of the Kai model (phenol concentrations: 100 g·m^−3^ (●), 150 g·m^−3^ (▲), 300 g·m^−3^ (■)).

**Figure 6:**
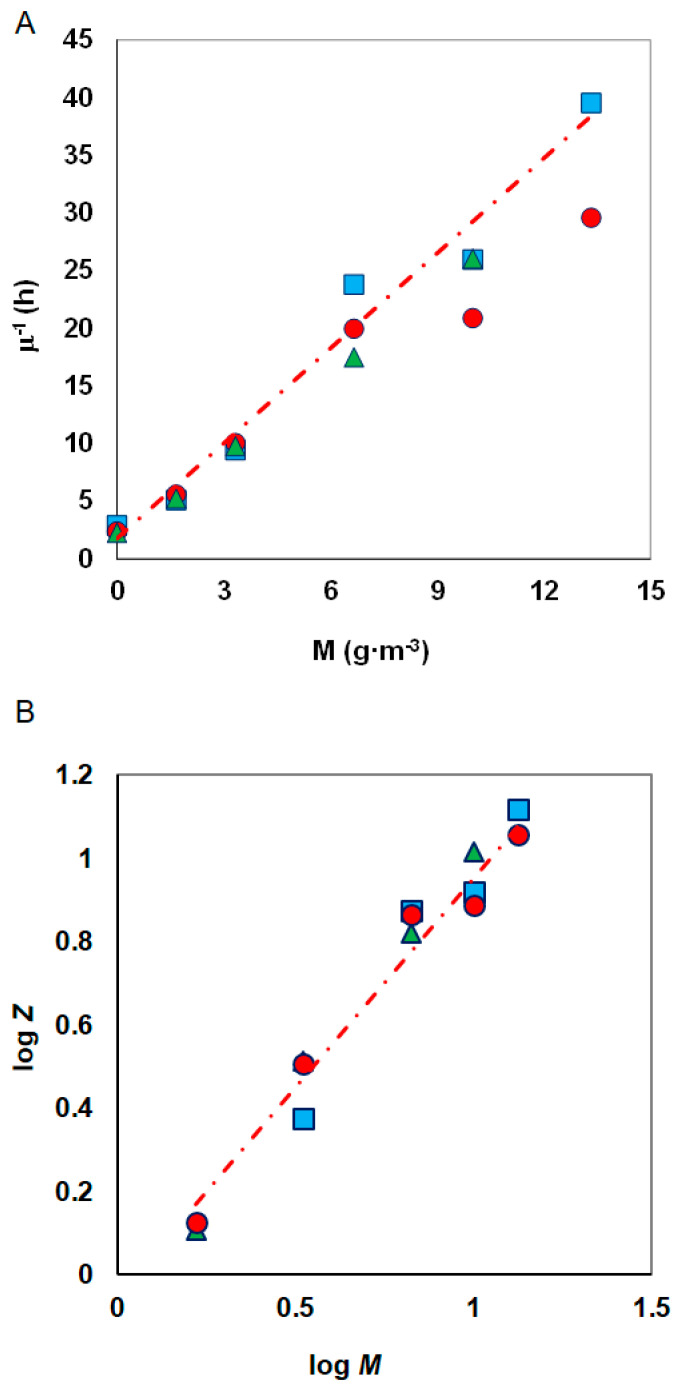
Method of determining the *K_IM_* constant according to (**A**) Amor et al. and (**B**) Nakamura and Sawada; phenol concentrations: 100 g·m^−3^ (●), 150 g·m^−3^ (▲), 300 g·m^−3^ (■); Z = $\frac{\mu_{max}\cdot S}{\mu \left(K_{s}+S+\frac{S^{2}}{K_{IS}}\right)}-1$.

**Figure 7:**
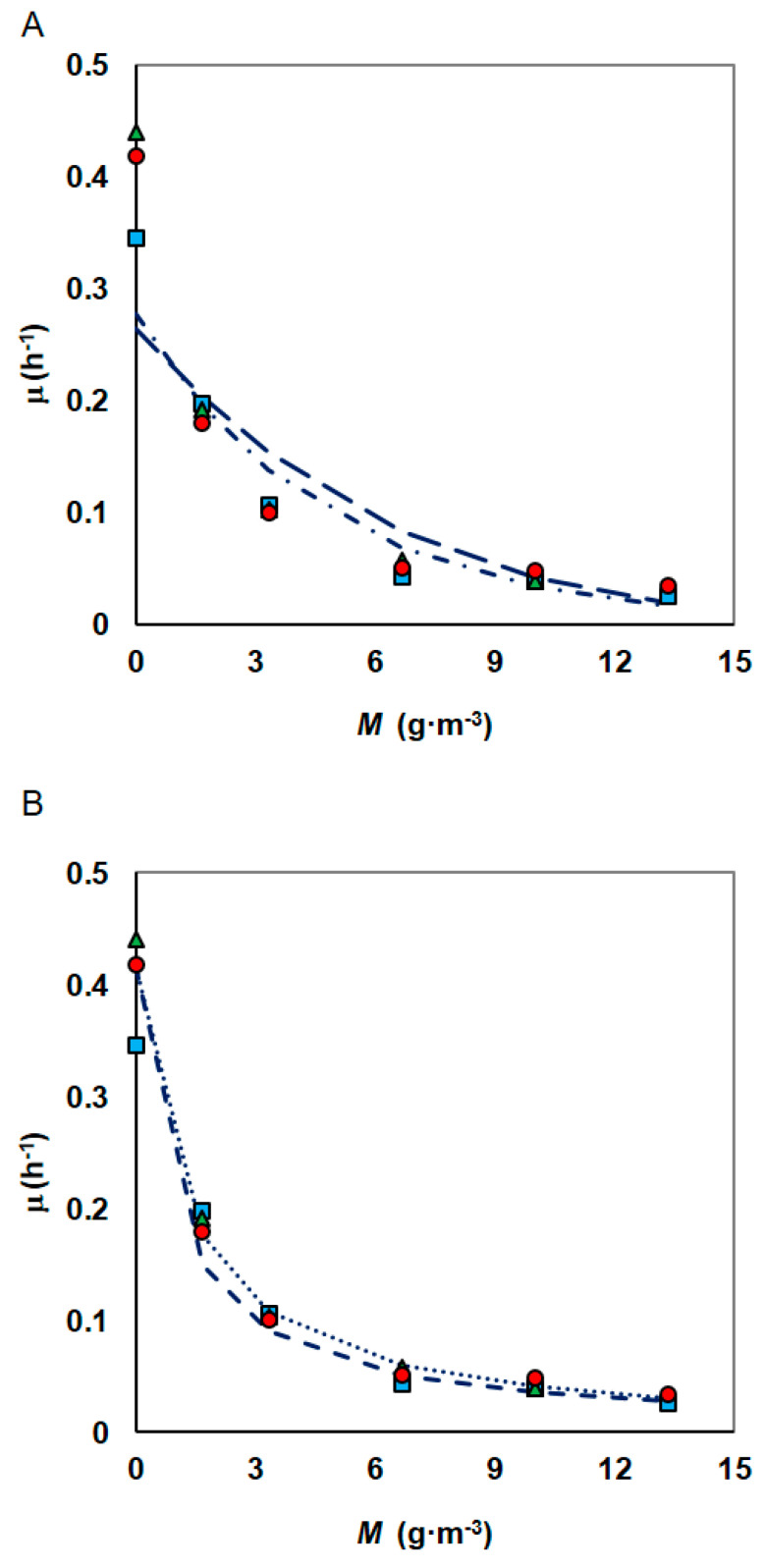
Inhibitory effect of increasing concentrations of nickel to the specific growth rate of *S. maltophilia* KB2 strain as fitted by the: (**A**) Han–Levenspiel and Kai models, (**B**) Andrews model with heavy metal inhibition (phenol concentrations: 100 g·m^−3^ (●), 150 g·m^−3^ (▲), 300 g·m^−3^ (■)).

Despite the growing number of studies presenting the results of research related to the hydrocarbons biodegradation in the presence of heavy metals, only a few authors propose a mathematical approach to this phenomenon. To study the kinetics of heavy metal inhibition, the experiments of the phenol biodegradation process for different nickel concentrations and constant phenol concentration were done. For every batch culture, the specific growth rate was determined and the created experimental database was used to estimate the parameters of selected mathematical models presented in Section 2.6. As shown in Figure 4, Figure 5, Figure 6 and Figure 7, also in these series of studies, the phenol concentration had less influence on the specific growth rate than metal presence.

#### 3.4.1. First Model

Han and Levenspiel proposed a generalization of the Monod equation with regard to inhibition effects caused by high substrate concentration or product or cell or other inhibitory substance [49]. To predict the impact of metal on organic biodegradation the modified Han–Levenspiel model (Equation (4)) is more often used. In this study, the value of *M_crit_* was taken to be equal to 33.3 g·m^−3^ based on the results of the tests described in Section 3.2.1. Then the constants of this modified equation μ_max_ and n could be determined from the plot $\log\mu =f(log(1-\frac{M}{M_{crit}})),$ Figure 4. The parameters μ_max_ and n obtained from the fitting data were 0.2647 h^−1^ and 5.1975 m^3^·g^−1^, respectively.

#### 3.4.2. Second Model

The specific growth rate at various concentrations of Ni was modelled using an exponential decay model [53]. In the graph ln *μ* = f (*M*), the measurement points are arranged along a straight line and its equation allows us to determine the values of the model constants (Figure 5). The parameters *μ_max_* and *k* obtained from the fitting data were 0.2778 h^−1^ and −0.21075, respectively.

#### 3.4.3. Third Model

The third model considers the metal inhibition by incorporating the metal inhibition constant (*K_IM_*) to the conventional growth model. This model is the product of two factors, the first one takes into account the substrate inhibition and the second one—metal influence. In the present study, Andrews model was selected to describe the substrate inhibition effect. The second factory of the Equation (6) includes the inhibitory effect of nickel. Two methods of determining the value of the metal inhibition constant *K_IM_* have been found in available publications [46,47]:

- according to Amor

The metals inhibition constant (*K_IM_*) was determined from the plot (specific growth rate)^−l^ versus heavy metal concentrations, according to the Equation (8) (Figure 6A).

- according to Nakamura

Constants *K_IM_* and *n* were estimated from the graph $\log\left[\frac{\mu_{max}\cdot S}{\mu \left(K_{s}+S+\frac{S^{2}}{K_{IS}}\right)}-1\right]=f\left(\log M\right)$ on which the results of experiments conducted for constant substrate concentration and different metal concentrations were plotted (Figure 6B).

The specific growth rate at various concentrations of nickel was modelled using three different equations for inhibitory effects of metal ion. Analysed models were able to fit the curve well (Figure 7) and the inhibition parameters obtained as per analysed models are tabulated in Table 3. Both the Kai and modified Han–Levenspiel models show good fitting (R^2^ = 0.937 and R^2^ = 0.904, respectively), but the third model shows even better fitting regardless of how *K_IM_* was determined. The results of the statistical analysis indicate that the third model with constants determined according to Nakamura’s guidelines was slightly more accurate one with coefficient R^2^ = 0.995.

Only several publications about the influence of the nickel presence on the phenol biodegradation process have appeared. Typically, the percent of substrate degradation or the biomass growth obtained for one nickel ions concentration were reported. Unfortunately, studies were not usually carried out for a wider concentration range, hence kinetic models could not be developed. Despite the significance of the heavy metal co-contamination studies, the use of a model involving metals as co-contamination is poorly studied and represented in the literature. To our knowledge, none of the literature is available to address the model for phenol biodegradation in the presence of nickel. In the previously cited works, when phenol was a substrate, another metal was an accompanying impurity or when the studies included nickel, another hydrocarbon was the carbon source. Nakamura and Sawada proposed a microbial growth model for the biodegradation of phenol in solution containing a heavy metal such as zinc or copper ions [46]. Whereas, in the work by Amor et al., the values of *K_I_* constants, estimated for the cultures containing nickel in the range 0.4 to 1 mM, were given [47]. Depending on the used carbon source, their values slightly changed, and so for toluene *K_I_* = 0.606 mM, for ethylbenzene *K_I_* = 0.690 mM and for o-xylene *K_I_* = 0.620 mM.

## 4. Conclusions

Chemical diversity of contaminations introduced daily into the natural environment is so huge that it is necessary to study the mutual influence of organic and inorganic compounds on efficiency of their microbial decomposition. In the discussed research, it was only found to have an inhibitory effect on the growth of tested bacteria, and at concentration higher than 33.3 g·m^−3^, their growth was negligible relative to inoculum size. Therefore, both phenol and nickel are factors that inhibit cell growth of the strain *Stenotrophomonas maltophilia* KB2.

Consequently, the studies to assess the growth of *S. maltophilia* KB2 in phenol and nickel co-contaminated media were carried out in several series. For phenol concentrations ranging from 0 to 300 g·m^−3^ and constant Ni concentration, such a strong metal inhibition effect was observed that it was not possible to confirm the substrate inhibition. In this case, the dependence of the specific growth rate on the initial phenol concentration is very well described by the Monod equations. The results of tests for varying metal concentrations (1.67–13.33 g·m^−3^) showed that the specific growth rate of *S.maltophilia* KB2 was strongly dependent on the nickel concentration in the culture medium. The best agreement between experiment and modelling was achieved using modified Andrews equation with Pearson correlation coefficient R^2^ equal to 0.995. The presented studies have attested that also in the presence of nickel the selected strain was able to phenol decomposition. Similar tests will be carried out for other metals (e.g., Zn, Cu), which will allow us to assess the universality of the presented procedure.

## Figures and Tables

**Table 1:** Comparison of the specific growth rate of different microbial strain.

Strain	*µ_max_* Parameter of Andrews Model (h^−1^)	*K_S_* (g·m^−3^)	*K_IS_* (g·m^−3^)	µ_max_* True Maximum Growth Rate (h^−1^)	Ref.
Mixed culture (*Stenotrophomonas* sp. N5, Advenella sp. B9)	0.08098	188.1	965.3	0.043	[55]
*Pseudomonas putida* ATCC17484	0.45	221.4	310.5	0.17	[56]
*Rhodococcus* sp. SKC	0.3	36.4	418.8	0.175	[16]
Activated sludge	0.4039	5.393	550.8	0.34	[57]
*Glutamibacter nicotianae* MSSRFPD35	0.574	20.29	268.1	0.375	[28]
*Stenotrophomonas maltophilia* KB2	1.584	185.4	106.1	0.43	This study

**Table 2:** Parameters of the equations for bacterial growth on phenol in the presence of nickel.

Nickel Concentration (g·m^−3^)	*µ_max_* (h^−1^)	*K_S_* (g·m^−3^)	*K_IS_* (g·m^−3^)	R^2^
0	1.584	185.4	106.1	0.9947
1.667	0.1999	6.2235	-	0.947
3.333	0.1089	5.6025	-	0.9931

**Table 3:** Comparison of 3 models of bacterial growth on phenol in the presence of nickel.

Equation Number	*μ_max_* (h^−1^)	*K_S_* (g·m^−3^)	*K_IS_* (g·m^−3^)	*K_IM_* (g·m^−3^)	*M_crit_* (g·m^−3^)	*k*	*n*	R^2^
(4)	0.2647				33.3		5.1975	0.904
(5)	0.2778					−0.2107		0.937
(6)	1.584	185.367	106.137	0.918			1.0	0.988
(6)	1.584	185.367	106.137	1.249			1.0706	0.995

## Data Availability

Not applicable.
